# The Effect of Smoking and Sex on the Association Between Long-term Alcohol Consumption and Metabolic Syndrome in a Middle-aged and Older Population

**DOI:** 10.2188/jea.JE20190328

**Published:** 2021-04-05

**Authors:** Kyueun Lee, Edward L Giovannucci, Jihye Kim

**Affiliations:** 1 Department of Medical Nutrition, Graduate School of East-West Medical Science, Kyung Hee University, Yongin, Republic of Korea; 2 Departments of Epidemiology and Nutrition, Harvard T.H. Chan School of Public Health and Harvard Medical School, Boston, MA, USA

**Keywords:** long-term alcohol consumption, metabolic syndrome, smoking status, hypertriglyceridemia, prospective study

## Abstract

**Background:**

The effect of smoking and sex on the relationship between alcohol consumption and risk of developing metabolic syndrome (MetS) and its components has not been investigated.

**Methods:**

A total of 5,629 Korean adults aged 40–69 years without MetS were recruited at baseline. Alcohol consumption was assessed biennially, and participants were classified as never, light, moderate, or heavy drinkers. Smoking status was examined at baseline and categorized into non-smokers and current smokers. Risk of incident MetS and its components according to alcohol consumption was examined by smoking status and sex using a multivariate Cox proportional hazards model.

**Results:**

During a follow-up of 12 years, 2,336 participants (41.5%) developed MetS. In non-smokers, light or moderate alcohol drinkers had a lower risk of developing MetS, abdominal obesity, hyperglycemia, hypertriglyceridemia, and low HDL-C compared with never drinkers. Heavy alcohol consumption was associated with a higher risk of incident elevated blood pressure (hazard ratio [HR] 1.48; 95% confidence interval [CI], 1.07–2.06; *P* = 0.020) in men and abdominal obesity (HR 1.86; 95% CI, 1.06–3.27; *P* = 0.030) in women. However, in smokers, the inverse association of light or moderate alcohol consumption with hypertriglyceridemia and abdominal obesity was not present, whereas a positive association between heavy alcohol consumption and hyperglycemia (HR 1.39; 95% CI, 1.07–1.80; *P* = 0.014) was observed.

**Conclusions:**

Smoking status and sex strongly affects the association between long-term alcohol consumption and MetS and its components by the amount of alcohol consumed.

## INTRODUCTION

Metabolic syndrome (MetS) is a complex of metabolic abnormalities, including abdominal obesity, hyperglycemia, hypertriglyceridemia, low levels of high-density lipoprotein cholesterol (HDL-C), and elevated blood pressure.^1^ MetS is considered a risk factor for development of cardiovascular disease (CVD) and type 2 diabetes mellitus, as well as for all-cause mortality.^2^ MetS prevalence increased in the American population from 32.9% to 34.7% between 2003–2004 and 2011–2012.^3^ A recent systemic review found that at least one-fifth of the adult population in the Asia-Pacific region had MetS.^4^

Lifestyle factors, such as alcohol drinking and smoking, are known to be major risk factors for MetS and its components. Evidence has shown that the association between alcohol consumption and MetS is complex and controversial because the association is reported to differ depending on the amount of alcohol consumed. Light alcohol consumption was inversely associated with MetS and heavy alcohol consumption was positively associated with MetS and its components.^5^^–^^7^ Some studies reported no significant association^8^^,^^9^ or positive association^10^ between alcohol consumption and MetS. Interactions with cigarette smoking, which tends to be correlated with alcohol intake, may contribute to the conflicting results. Recent studies showed that it is important to account for smoking status when assessing preventability of metabolic diseases.^11^^,^^12^ Several studies have shown the association of cigarette smoking and alcohol consumption with MetS.^13^^–^^15^ However, the interrelationship of alcohol drinking and smoking habit on MetS and its components have not been investigated in the prospective design.

Furthermore, the influence of alcohol consumption on risk of MetS and its components may vary across ethnicity.^16^ Alcohol may be more sensitive in Asian population than Caucasian due to genetic difference in alcohol-metabolizing enzymes.^17^ Therefore, the association of long-term alcohol consumption and smoking with metabolic risk factors could be different in Asian populations.

In this context, this study explored the prospective association between long-term alcohol consumption and risk of MetS and its components by smoking status and sex in middle-aged and older adults using data from a large community-based cohort study.

## METHODS

### Subject population

Data from the Korean Genome and Epidemiology Study (KoGES) was used for this study. KoGES is an ongoing prospective cohort study to explore genetic, diet, and environmental factors related to CVD and diabetes mellitus in the Korean general population.^18^ A total of 10,030 participants, aged 40–69 years, living in Ansan and Ansung City were recruited between 2001 and 2002 at baseline. Questionnaires collected demographic information, medical history, and lifestyle factors at baseline. Anthropometric measurements and biochemical data for MetS diagnosis were collected biennially from 2003 to 2014.

Among the 10,030 participants, exclusion criteria included: had MetS or its components at baseline, refused to participate in follow-up examinations, had CVD or cancer at baseline, did not complete the alcohol consumption questionnaire, were missing information on covariates, or were past drinkers. Included in the analyses were 5,629 participants (2,884 men and 2,745 women) for MetS, 4,912 participants for abdominal obesity, 6,528 participants for hyperglycemia, 5,064 participants for hypertriglyceridemia, 4,996 participants for low HDL cholesterol, and 4,514 participants for elevated blood pressure (eFigure 1).

This study has been carried out in accordance with Declaration of Helsinki. The study protocol was approved by the Institutional Review Boards of the Korea Centers for Disease Control and Prevention and Kyung Hee University (KHSIRB-16-022). Written informed consent was obtained from all participants.

### Definition of MetS

MetS incidence was measured biennially. The diagnostic criteria for MetS were presence of three or more of^19^: (1) abdominal obesity, defined as waist circumference ≥90 cm in men or ≥80 cm in women; (2) hyperglycemia, defined as fasting blood glucose ≥100 mg/dL, current use of insulin or oral hypoglycemic medication, or physician’s diagnosis; (3) hypertriglyceridemia, defined as plasma triglyceride (TG) concentration ≥150 mg/dL; (4) low HDL-C, defined as plasma HDL-C concentration <40 mg/dL in men or <50 mg/dL in women; and (5) elevated blood pressure, defined as systolic blood pressure (SBP) ≥130 mm Hg, diastolic blood pressure (DBP) ≥85 mm Hg, use of antihypertensive medication, or physician’s diagnosis of hypertension.

### Alcohol consumption

Alcohol consumption was biennially evaluated via questionnaire-based interview using a standardized manual. Alcohol consumption was asked about with question “How often did you drink alcohol during the past year?” For the assessment of habitual alcohol intake, alcohol consumption was calculated as the average of baseline and all follow-up examinations right before the MetS was assessed.

Six types of alcoholic beverages, including traditional beverages (such as soju, chungju, and makgeolli), beer, wine, and hard liquor were examined. Alcohol consumption was evaluated among current and former drinkers who consumed alcohol within 1 year. Participants were asked to state the average frequency and portion size of alcohol drinks during the past year. Frequency had six options (once/month, 2–3 times/month, once/week, 2–3 times/week, 4–6 times/week, once/day). Portion size of drinks was determined with an open-ended question. The volume of one standard drink was described in the question as depending on alcohol type (beer: 220 cc, wine: 90 cc, hard liquor: 30 cc, soju: 50 cc, chungju: 50 cc, makgeolli: 240 cc). Consumption of each drink was calculated as frequency of alcohol drink multiplied by portion size and converted to alcohol consumption. Specific alcohol consumption in g/d was calculated based on alcohol content (4.5% beer, 12% wine, 40% hard liquor, 22% soju, 16% chungju, and 6% makgeolli). Total alcohol consumption was calculated by summing alcohol consumption from six alcohol drinks. Never drinkers are individuals who had never drank during life time. Past drinkers are individuals who stopped drinking alcohol before two years at each examination were excluded from the analysis because of small number (*n* = 127). Alcohol consumption was categorized into four groups based on World Health Organization criteria^20^ as: (1) never drinker; (2) light (0–15 g/day men, 0–10 g/day women); (3) moderate (15<–40 g/day men, 10<–20 g/day women); and (4) heavy (>40 g/day men, >20 g/day women).

### Anthropometric measurements and biochemical assessments

Health examinations were conducted by trained research staff using a standardized protocol. Height and body weight were measured at baseline while participants wore a thin cloth and socks without shoes. Body mass index (BMI) was calculated as body weight (kg) divided by the square of height (m^2^). Waist circumference was measured at the midpoint between the lowest rib and the iliac crest and the average of three repeated measurements was used. Blood pressure was measured using a mercury sphygmomanometer (W.A Baum Co. Inc., Copiague, NY, USA) with patients in a sitting position after 5 minutes of relaxing. SBP and DBP were measured twice at phase I Korotkoff sound for SBP and phase V for DBP and reported as the average of both arm readings. Blood samples were collected after at least 8 hours fasting and plasma was separated for biochemical measurements. Plasma concentrations of glucose, TG, and HDL-C were enzymatically measured using an autoanalyzer (ADIVA 1650, Bayer HealthCare, Tarrytown, NY, USA).

### Covariates

Data on demographic characteristics, socioeconomic status, and lifestyle factors were examined at baseline using structured questionnaires.^21^ Monthly household income was categorized into four groups: <$890 (approximately 1 million Korean won in 2018), $890 to <$1,780, $1,780 to <$2,670, and ≥$2,670. Educational level was categorized into three groups; ≤6, 7 to ≤12, and >12 years. Smoking status was categorized into three groups: never, former, and current smoker. Non-smoker included never smoker and former smoker. Former and current smokers were asked about smoking duration. Physical activity was evaluated using metabolic equivalent of task-hours per day.^22^ Participants were asked about hours spent on sleep and activities classified according to intensity: sedentary, very light, light, moderate, and heavy activity.

Dietary intake was assessed at baseline and second follow-up examination (2005–2006) with a validated semi-quantitative food frequency questionnaire (FFQ).^23^ For participants who developed MetS or were censored between baseline and second follow-up, food consumption was evaluated based on FFQ at baseline. For those who developed MetS or were censored after second follow-up, food consumption was calculated based on the average of FFQs at baseline and second follow-up. Intake of 12 types of fruit, meat (pork, beef, chicken, and processed meat), refined grains (white rice, noodles, and breads), whole grains (barley, multigrain, and mixed grain powder), and dairy foods (including milk and yogurt) were evaluated. Nutrient intake was calculated using a food composition table provided by Korean Nutrition Society.^24^

### Statistical analysis

Characteristics of participants at baseline are expressed as mean and standard deviation (continuous variables) or number and percentage (categorical variables). Differences in characteristics according to development of MetS were examined using chi-square tests for categorical variables and Student’s *t*-tests for continuous variables. Comparisons of variables across four categories of alcohol consumption were made using either chi-square tests or generalized linear models, as appropriate. Hazard ratios (HRs) and 95% confidence intervals (CIs) for risk of MetS and its components according to alcohol consumption were calculated using multivariate Cox proportional hazards models. Alcohol drinking status was categorized into four groups and never drinkers were the reference group. Three models were fitted as: model 1 adjusted for age; model 2 adjusted for age, residential location, household income, education level, smoking status, physical activity, menopause (for women), and BMI; and model 3 adjusted for all covariates in model 2 plus energy intake and food intake (fruits, meat, refined grain, whole grain, and dairy products). To select variables for adjustment in the multivariable model, potential confounders from the literature were accounted for with statistical approaches, such as stepwise procedures or comparing adjusted and unadjusted effect estimates of potential confounders.^25^ Tests for linear trends were based on the median value of each category. Stratified analysis was conducted because of interactions between alcohol consumption and sex (*P* < 0.0001) or smoking status (non-smoker/current smoker) or residential location (rural/urban). Logistic regression analysis was used to examine the effect of interaction between alcohol consumption and smoking status on hypertriglyceridemia by the category of alcohol consumption.

The proportional hazard assumption was confirmed graphically using log–log plots and statistically using Schoenfeld’s residuals^26^ with no violation of the assumption. Person-year was estimated as the actual time at risk-between the start date of study and the end date of study (date of disease incidence, date of loss to follow-up, date of end of study), in days, that all participants contributed to a study, and then converted to years. All data were analyzed using SAS software, version 9.4 (SAS Institute, Cary, NC, USA). *P* < 0.05 was considered statistically significant for two-sided tests.

## RESULTS

The follow-up rate was 73% for 42,785 person-years. The average follow-up period was 91 months (range, 17–151 months). A total of 2,336 (41.5%) participants acquired MetS during the follow-up period.

Baseline characteristics of the study population according to incidence of MetS by sex are in Table 1. Men who developed MetS were more likely to live in rural areas and to be current smokers. They had higher BMI and higher intake of total energy, meat, refined grains. They had less intake of whole grains and dairy products compared to those who did not develop MetS. Women who developed MetS were older and more likely to live in rural areas. They had lower income, were less educated and had higher physical activity and BMI. They had higher intake of energy from carbohydrate and refined grains and lower intake of energy from protein and fat, fruits, meat, whole grains, and dairy products compared to those who did not develop MetS.

**Table 1.** Baseline characteristics of Korean adults according to development of metabolic syndrome

	MetS (*n* = 2,336)	Non-MetS (*n* = 3,293)	*P* value
Men (*n* = 2,884)			
*n* (%)	1,122 (38.9)	1,762 (61.1)	
Age, years	51.1 (8.3)	51.1 (8.9)	0.918
Area of residence, *n* (%)			<0.0001
Rural, Ansung	549 (48.9)	718 (40.7)	
Urban, Ansan	573 (51.1)	1,044 (59.3)	
Household income, *n* (%)			0.811
<$890/month	301 (26.8)	463 (26.3)	
$890 to <$1,780	346 (30.8)	532 (30.2)	
$1,780 to <$2,670	220 (19.6)	372 (21.1)	
≥$2,670	255 (22.8)	395 (22.4)	
Education level, *n* (%)			0.400
≤6 years	207 (18.5)	348 (19.8)	
7 to ≤12 years	671 (59.8)	1,009 (57.2)	
>12 years	244 (21.7)	405 (23.0)	
Smoking status, *n* (%)			0.006
Never smokers	199 (17.7)	384 (21.8)	
Former smokers	324 (28.9)	534 (30.3)	
Current smokers	599 (53.4)	844 (47.9)	
Physical activity			
MET, hours/day	24.8 (15.8)	24.4 (15.4)	0.502
BMI, kg/m^2^	24.6 (2.5)	22.9 (2.6)	<0.0001
Alcohol intake, %			<0.0001
Never	211 (18.8)	297 (16.9)	
Light	369 (32.9)	781 (44.3)	
Moderate	334 (29.8)	449 (25.5)	
Heavy	208 (18.5)	235 (13.3)	
Total energy intake, kcal/day	2,065 (753)	2,000 (616)	0.015
Percent from energy, %			
Carbohydrates	70.4 (7.3)	70.6 (6.9)	0.344
Protein	13.9 (2.4)	13.7 (5.1)	0.163
Fat	15.8 (5.4)	15.7 (5.1)	0.508
Food intake, servings/day			
Fruits	2.5 (2.3)	2.6 (2.1)	0.107
Meat	0.7 (0.7)	0.6 (0.5)	0.003
Refined grains	2.3 (1.6)	2.2 (1.5)	0.031
Whole grains	1.6 (1.5)	1.7 (1.4)	0.117
Dairy	0.5 (0.6)	0.6 (0.6)	0.074

Women (*n* = 2,745)			
*n* (%)	1,214 (44.2)	1,531 (55.8)	
Age, years	53.1 (8.7)	48.3 (7.9)	<0.0001
Area of residence, *n* (%)			<0.0001
Rural, Ansung	724 (59.6)	495 (32.3)	
Urban, Ansan	490 (40.4)	1,036 (67.7)	
Household income, *n* (%)			<0.0001
<$890/month	529 (43.6)	389 (25.4)	
$890 to <$1,780	370 (30.5)	469 (30.6)	
$1,780 to <$2,670	174 (14.3)	357 (23.3)	
≥$2,670	141 (11.6)	316 (20.7)	
Education level, *n* (%)			<0.0001
≤6 years	568 (46.8)	401 (26.2)	
7 to ≤12 years	584 (48.1)	977 (63.8)	
>12 years	62 (5.1)	153 (10.0)	
Smoking status, *n* (%)			0.621
Never smokers	1,162 (95.7)	1,464 (95.6)	
Former smokers	14 (1.2)	13 (0.9)	
Current smokers	38 (3.1)	54 (3.5)	
Physical activity			
MET, hours/day	23.7 (15.1)	20.7 (13.1)	<0.0001
BMI, kg/m^2^	25.0 (3.0)	23.3 (2.8)	<0.0001
Alcohol intake, %			<0.0001
Never	753 (62.0)	806 (52.7)	
Light	414 (34.1)	662 (43.2)	
Moderate	36 (3.0)	42 (2.7)	
Heavy	11 (0.9)	21 (1.4)	
Total energy intake, kcal/day	1,912 (785)	1,901 (696)	0.692
Percent from energy, %			
Carbohydrates	73.1 (7.2)	71.3 (7.1)	<0.0001
Protein	13.4 (2.3)	13.8 (2.3)	<0.0001
Fat	13.5 (5.4)	14.9 (5.3)	<0.0001
Food intake, servings/day			
Fruits	3.2 (2.9)	3.5 (2.6)	0.019
Meat	0.4 (0.5)	0.5 (0.6)	0.016
Refined grains	1.6 (1.5)	1.4 (1.2)	<0.0001
Whole grains	1.8 (1.4)	1.9 (1.2)	0.013
Dairy	0.7 (0.7)	0.7 (0.7)	0.001

BMI, body mass index; MET, metabolic equivalent of task; MetS, metabolic syndrome.

Values are *n* (%) or means (standard deviation).

*P* values were assessed with Student’s *t*-test (continuous variables) or chi-square test (categorized variables).

Baseline characteristics of the study population according to alcohol consumption by sex are in Table 2. Men in the highest category of alcohol consumption were younger and more likely to live in urban areas. They were less likely to be educated, had higher income, and were more likely to be current smokers. They had higher physical activity and higher intake of total energy, and energy from protein, fat, and meat. They had lower intake of energy from carbohydrates and fruits compared with never drinkers. Participants in the highest category of alcohol consumption showed significantly higher levels of fasting glucose, TG, HDL-C, SBP, and DBP than never drinkers. Women in the highest category of alcohol consumption were younger, more likely to live in urban areas, and more likely to be educated. They had higher income, were more likely to be current smokers, had lower physical activity, had higher intake of energy from protein, fat, meat, and refined grains, and had lower intake of energy from carbohydrates and whole grains than never drinkers.

**Table 2.** Baseline characteristics of Korean adults according to alcohol consumption by sex

	Alcohol consumption (g/day)				

	Never	Light (0–15)	Moderate (15<–40)	Heavy (>40)	*P* trend
**Men**					
*n* (*n* of cases)	508 (211)	1,150 (369)	783 (334)	443 (208)	
Age, years	53.1 (9.0)^a^	51.1 (8.6)^b^	50.4 (8.5)^b^	50.1 (8.6)^b^	<0.0001
Residential location, %					0.006
Rural, Ansung	49.4	41.6	41.9	47.4	
Urban, Ansan	50.6	58.4	58.1	52.6	
Education level, %					<0.0001
≤6 years	23.4	15.7	20.1	22.4	
7 to ≤12 years	55.5	59.5	56.6	61.2	
>12 years	21.1	24.8	23.3	16.4	
Household income, %					<0.0001
<$890/month	33.5	23.3	24.9	29.6	
$890 to <$1,780	31.7	32.1	30.9	23.9	
$1,780 to <$2,670	15.6	22.3	19.4	23.7	
≥$2,670	19.3	22.3	24.8	22.8	
Smoking status, %					<0.0001
Never	33.7	23.3	12.4	10.6	
Former	28.2	31.6	29.9	26.6	
Current	38.2	45.1	57.7	62.8	
Physical activity					
MET, hours/day	24.5 (16.2)^a,b^	23.8 (15.2)^a^	24.4 (15.1)^a,b^	26.6 (16.5)^b^	0.017
BMI, kg/m^2^	23.5 (2.9)	23.5 (2.6)	23.7 (2.6)	23.5 (2.8)	0.224
Alcohol intake, g/day	0	5.1 (0.3–14.5)	23.7 (15.6–39.4)	50.9 (40.5–252.0)	
Nutrient intake					
Total energy, kcal/day	2,029 (773)^a,b^	1,996 (611)^a^	2,020 (575)^a,b^	2,107 (846)^b^	0.032
Carbohydrate, %	71.9 (7.4)^a^	70.9 (6.8)^b^	69.7 (6.7)^c^	69.3 (7.8)^c^	<0.0001
Protein, %	13.2 (2.4)^a^	13.6 (2.2)^a^	14.1 (2.1)^b^	14.1 (2.6)^b^	<0.0001
Fat, %	14.9 (5.4)^a^	15.5 (5.0)^b^	16.2 (5.0)^c^	16.4 (5.7)^c^	<0.0001
Food intake, servings/day					
Fruits	2.8 (3.0)^a^	2.7 (2.2)^a^	2.4 (1.7)^b^	2.3 (1.9)^b^	<0.0001
Meat	0.5 (0.6)^a^	0.6 (0.5)^a^	0.7 (0.5)^b^	0.8 (0.9)^c^	<0.0001
Refined grains	2.3 (1.6)^a,b^	2.2 (1.5)^a^	2.2 (1.6)^a,b^	2.4 (1.7)^b^	0.029
Whole grains	1.6 (1.5)	1.7 (1.5)	1.7 (1.4)	1.6 (1.5)	0.531
Dairy	0.5 (0.6)	0.6 (0.6)	0.5 (0.7)	0.5 (0.6)	0.063
Waist circumference, cm	81.3 (7.6)^a,b^	81.2 (6.8)^a^	82.1 (6.3)^b^	81.9 (6.6)^a,b^	0.029
Fasting glucose, mg/dL	90.7 (20.1)^a^	89.6 (15.3)^a^	91.6 (17.7)^a^	94.7 (23.9)^b^	<0.0001
Triglycerides, mg/dL	129.3 (78.6)^a^	136.7 (87.0)^a^	149.7 (110.2)^b^	169.2 (137.4)^c^	<0.0001
HDL-C, mg/dL	45.8 (9.5)^a^	48.7 (10.7)^b^	52.0 (11.7)^c^	54.2 (13.1)^d^	<0.0001
SBP, mm Hg	118.0 (15.7)^a,b^	117.6 (15.8)^a^	119.6 (16.5)^b,c^	121.7 (16.3)^c^	<0.0001
DBP, mm Hg	78.3 (10.4)^a^	79.3 (10.2)^a^	80.8 (10.5)^b^	82.1 (10.6)^b^	<0.0001
MetS status, %	41.5	32.1	42.7	47.0	<0.0001
	
	Never	Light (0–10)	Moderate (10<–20)	Heavy (>20)	*P* trend

**Women**					
*n* (*n* of cases)	1,559 (753)	1,076 (414)	78 (36)	32 (11)	
Age, years	52.2 (8.9)^a^	48.3 (7.7)^b^	47.2 (7.5)^b^	44.7 (4.8)^b^	<0.0001
Residential location, %					<0.0001
Rural, Ansung	48.6	39.8	33.3	25.0	
Urban, Ansan	51.4	60.2	66.7	75.0	
Education level, %					<0.0001
≤6 years	40.9	28.5	20.5	21.9	
7 to ≤12 years	51.1	63.8	73.1	68.8	
>12 years	8.0	7.7	6.4	9.3	
Household income, %					<0.0001
<$890/month	39.5	25.8	20.5	25.0	
$890 to <$1,780	29.1	33.0	32.1	18.8	
$1,780 to <$2,670	16.8	22.1	26.9	31.2	
≥$2,670	14.6	19.1	20.5	25.0	
Smoking status, %					<0.0001
Never	97.2	94.6	82.1	90.7	
Former	0.8	1.0	5.1	0	
Current	2.0	4.4	12.8	9.4	
Physical activity					
MET, hours/day	22.7 (14.5)	21.3 (13.7)	20.6 (10.1)	20.0 (14.3)	0.044
BMI, kg/m^2^	24.0 (3.1)	23.9 (2.9)	24.7 (3.0)	24.7 (2.9)	0.080
Alcohol intake, g/day	0	1.7 (0.2–9.8)	13.3 (10.4–19.4)	33.4 (20.3–70.3)	
Nutrient intake					
Total energy, kcal/day	1,887 (741)	1,930 (728)	1,904 (671)	2,009 (915)	0.422
Carbohydrate, %	73.1 (7.2)^a^	71.2 (6.8)^b^	68.2 (8.1)^c^	67.1 (8.3)^c^	<0.0001
Protein, %	13.4 (2.3)^a^	13.8 (2.2)^b^	14.7 (2.7)^c^	14.9 (3.2)^c^	<0.0001
Fat, %	13.5 (5.3)^a^	15.1 (5.1)^b^	17.2 (6.0)^c^	17.9 (5.7)^c^	<0.0001
Food intake, servings/day					
Fruits	3.2 (2.7)^a^	3.6 (2.7)^b^	3.2 (3.2)^a,b^	2.9 (2.6)^a,b^	0.009
Meat	0.4 (0.5)^a^	0.5 (0.6)^b^	0.6 (0.5)^b^	1.0 (1.2)^c^	<0.0001
Refined grains	1.6 (1.4)^a^	1.4 (1.2)^b^	1.4 (1.2)^b^	2.0 (1.2)^c^	0.035
Whole grains	1.9 (1.3)^a^	1.9 (1.3)^a^	1.8 (1.2)^a,b^	1.1 (1.1)^b^	0.004
Dairy	0.7 (0.7)	0.7 (0.7)	0.7 (0.7)	0.7 (0.7)	0.102
Waist circumference, cm	78.7 (8.9)^a^	77.3 (8.4)^b^	79.9 (9.0)^a,b^	78.7 (8.6)^a,b^	0.0003
Fasting glucose, mg/dL	86.3 (13.6)	85.9 (11.2)	87.5 (9.5)	87.7 (6.5)	0.589
Triglycerides, mg/dL	106.5 (54.9)^a^	99.4 (47.7)^b^	102.2 (44.8)^a,b^	110.2 (40.7)^a,b^	0.006
HDL-C, mg/dL	54.2 (11.4)	55.2 (11.3)	57.2 (12.0)	57.9 (10.3)	0.007
SBP, mm Hg	116.4 (17.9)^a^	112.4 (15.9)^b^	114.1 (15.1)^a,b^	110.8 (15.2)^a,b^	<0.0001
DBP, mm Hg	76.1 (10.9)^a^	74.4 (10.0)^b^	76.4 (9.4)^a,b^	75.4 (9.7)^a,b^	0.001
MetS status, %	48.3	38.9	46.2	34.4	<0.0001

BMI, body mass index; DBP, diastolic blood pressure; HDL-C, high density lipoprotein cholesterol; MET, metabolic equivalent of task; MetS, metabolic syndrome; SBP, systolic blood pressure.

Values are % or means (standard deviation) except for alcohol intake (median (ranges)).

*P* trend was assessed by generalized linear models or chi-square test.

^a,b,c,d^Differences of variables across alcohol consumption were examined by post hoc test (Tukey’s test for multiple comparisons). Unlike superscripts mean significant differences across alcohol consumption.

HRs and 95% CIs for incident MetS and components of MetS according to alcohol consumption by sex are in Table 3. Non-linear association between alcohol consumption and incidence of MetS was observed (*P_non-linearity_* = 0.04 in men, *P_non-linearity_* = 0.02 in women). Light alcohol consumption (≤15 g/day in men, ≤10 g/day in women) was associated with a lower risk of incident MetS (HR 0.65; 95% CI, 0.55–0.77; *P* < 0.0001 for men and HR 0.77; 95% CI, 0.68–0.87; *P* < 0.0001 women) and all components of MetS in both men and women after adjustment for potential confounders. Moderate alcohol consumption (15–40 g/day in men, 10–20 g/day in women) was associated with a lower risk of incident MetS, abdominal obesity and low HDL-C in men, and low HDL-C in women. Heavy alcohol consumption (>40 g/day for men, >20 g/day for women) was associated with a greater risk of hyperglycemia (HR 1.28; 95% CI, 1.06–1.54; *P* = 0.010) and elevated blood pressure (HR 1.49; 95% CI, 1.21–1.85; *P* = 0.0002) and a lower risk of low HDL-C (HR 0.45; 95% CI, 0.37–0.54; *P* < 0.0001) in men only. For women, heavy alcohol consumption was associated with a greater risk of abdominal obesity (HR 1.77; 95% CI, 1.03–3.05; *P* = 0.038). No association was seen between heavy alcohol consumption and risk of incident MetS regardless of sex.

**Table 3.** Hazard ratios and 95% confidence intervals for risk of incident metabolic syndrome components according to alcohol consumption

	Alcohol consumption (g/day)				

	Never	Light (0–15)	Moderate (15<–40)	Heavy (>40)	*P* trend
Men					
Abdominal obesity (*n* = 3,040)					
*n* (*n* of cases)	539 (158)	1,224 (330)	829 (256)	448 (145)	
Model 1^a^	1	0.71 (0.59–0.85)	0.81 (0.67–0.99)	0.95 (0.76–1.18)	0.809
Model 2^b^	1	0.72 (0.60–0.87)	0.78 (0.64–0.95)	0.90 (0.72–1.12)	0.616
Model 3^c^	1	0.72 (0.60–0.86)	0.75 (0.62–0.91)	0.84 (0.67–1.05)	0.232
Hyperglycemia (*n* = 2,941)					
*n* (*n* of cases)	546 (241)	1,221 (444)	764 (371)	410 (237)	
Model 1	1	0.69 (0.58–0.80)	1.02 (0.87–1.21)	1.44 (1.21–1.73)	<0.0001
Model 2	1	0.68 (0.58–0.80)	0.99 (0.84–1.17)	1.37 (1.13–1.65)	<0.0001
Model 3	1	0.69 (0.59–0.81)	0.95 (0.81–1.13)	1.28 (1.06–1.54)	<0.0001
Hypertriglyceridemia (*n* = 2,130)					
*n* (*n* of cases)	439 (181)	873 (327)	537 (262)	281 (143)	
Model 1	1	0.76 (0.63–0.91)	1.06 (0.88–1.28)	1.25 (1.00–1.55)	0.001
Model 2	1	0.74 (0.62–0.89)	0.97 (0.80–1.18)	1.12 (0.89–1.40)	0.042
Model 3	1	0.74 (0.62–0.89)	0.94 (0.77–1.14)	1.05 (0.84–1.32)	0.164
Low HDL-C (*n* = 2,887)					
*n* (*n* of cases)	458 (290)	1,043 (512)	872 (390)	514 (230)	
Model 1	1	0.60 (0.52–0.69)	0.51 (0.44–0.59)	0.54 (0.45–0.64)	<0.0001
Model 2	1	0.55 (0.48–0.64)	0.45 (0.39–0.53)	0.46 (0.39–0.56)	<0.0001
Model 3	1	0.55 (0.48–0.64)	0.44 (0.38–0.52)	0.45 (0.37–0.54)	<0.0001
Elevated blood pressure (*n* = 2,031)					
*n* (*n* of cases)	395 (187)	859 (367)	492 (232)	285 (179)	
Model 1	1	0.80 (0.67–0.96)	0.92 (0.76–1.12)	1.61 (1.31–1.98)	<0.0001
Model 2	1	0.82 (0.68–0.97)	0.93 (0.76–1.13)	1.55 (1.25–1.92)	<0.0001
Model 3	1	0.82 (0.68–0.98)	0.90 (0.74–1.10)	1.49 (1.21–1.85)	0.0002
Metabolic syndrome (*n* = 2,884)					
*n* (*n* of cases)	508 (211)	1,150 (369)	783 (334)	443 (208)	
Model 1	1	0.66 (0.56–0.78)	0.93 (0.78–1.10)	1.15 (0.95–1.40)	0.001
Model 2	1	0.65 (0.55–0.77)	0.87 (0.73–1.03)	1.12 (0.92–1.36)	0.007
Model 3	1	0.65 (0.55–0.77)	0.85 (0.71–1.01)	1.08 (0.88–1.31)	0.030
	
	Never	Light (0–10)	Moderate (10<–20)	Heavy (>20)	*P* trend

Women					
Abdominal obesity (*n* = 1,872)					
*n* (*n* of cases)	1,079 (586)	727 (343)	39 (20)	27 (14)	
Model 1	1	0.78 (0.68–0.89)	1.02 (0.65–1.60)	1.28 (0.75–2.18)	0.027
Model 2	1	0.83 (0.72–0.95)	1.28 (0.81–2.00)	1.86 (1.09–3.19)	0.403
Model 3	1	0.83 (0.72–0.95)	1.34 (0.85–2.10)	1.77 (1.03–3.05)	0.437
Hyperglycemia (*n* = 3,587)					
*n* (*n* of cases)	2,109 (694)	1,352 (392)	87 (30)	39 (15)	
Model 1	1	0.86 (0.76–0.97)	1.27 (0.88–1.84)	1.87 (1.12–3.13)	0.686
Model 2	1	0.87 (0.76–0.98)	1.09 (0.75–1.59)	1.73 (1.03–2.90)	0.514
Model 3	1	0.87 (0.76–0.98)	1.03 (0.71–1.50)	1.61 (0.96–2.70)	0.400
Hypertriglyceridemia (*n* = 2,934)					
*n* (*n* of cases)	1,716 (746)	1,112 (379)	68 (24)	38 (18)	
Model 1	1	0.68 (0.60–0.77)	0.81 (0.54–1.22)	1.27 (0.79–2.03)	<0.0001
Model 2	1	0.69 (0.61–0.79)	0.77 (0.51–1.17)	1.20 (0.75–1.93)	<0.0001
Model 3	1	0.70 (0.62–0.79)	0.76 (0.50–1.15)	1.11 (0.69–1.78)	<0.0001
Low HDL-C (*n* = 2,109)					
*n* (*n* of cases)	1,302 (961)	707 (421)	52 (30)	48 (30)	
Model 1	1	0.60 (0.53–0.67)	0.66 (0.46–0.95)	0.76 (0.53–1.09)	<0.0001
Model 2	1	0.60 (0.53–0.67)	0.64 (0.45–0.93)	0.76 (0.52–1.09)	<0.0001
Model 3	1	0.60 (0.53–0.68)	0.63 (0.44–0.91)	0.69 (0.48–1.00)	<0.0001
Elevated blood pressure (*n* = 2,483)					
*n* (*n* of cases)	1,404 (619)	991 (346)	58 (24)	30 (12)	
Model 1	1	0.82 (0.72–0.94)	1.22 (0.81–1.84)	1.15 (0.65–2.04)	0.124
Model 2	1	0.84 (0.73–0.96)	1.17 (0.77–1.77)	1.19 (0.67–2.12)	0.183
Model 3	1	0.84 (0.73–0.96)	1.11 (0.73–1.68)	1.12 (0.63–1.99)	0.123
Metabolic syndrome (*n* = 2,745)					
*n* (*n* of cases)	1,559 (753)	1,076 (414)	78 (36)	32 (11)	
Model 1	1	0.76 (0.67–0.86)	1.08 (0.77–1.51)	1.06 (0.58–1.92)	0.004
Model 2	1	0.77 (0.68–0.87)	0.96 (0.69–1.35)	1.09 (0.60–1.98)	0.003
Model 3	1	0.77 (0.68–0.87)	0.92 (0.66–1.29)	0.98 (0.54–1.79)	0.002

HDL-C, high density lipoprotein cholesterol.

HRs and 95% CIs for metabolic syndrome and components were obtained using a multivariate Cox proportional hazards model.

^a^Model 1 was adjusted for age.

^b^Model 2 was adjusted for age, residential location, household income, education level, smoking status, menopausal status (for women), physical activity, and body mass index.

^c^Model 3 was adjusted for age, residential location, household income, education level, smoking status, menopausal status (for women), physical activity, body mass index, energy intake, fruit intake, meat intake, refined grain intake, whole grain intake, and dairy intake.

**Figure 1.**
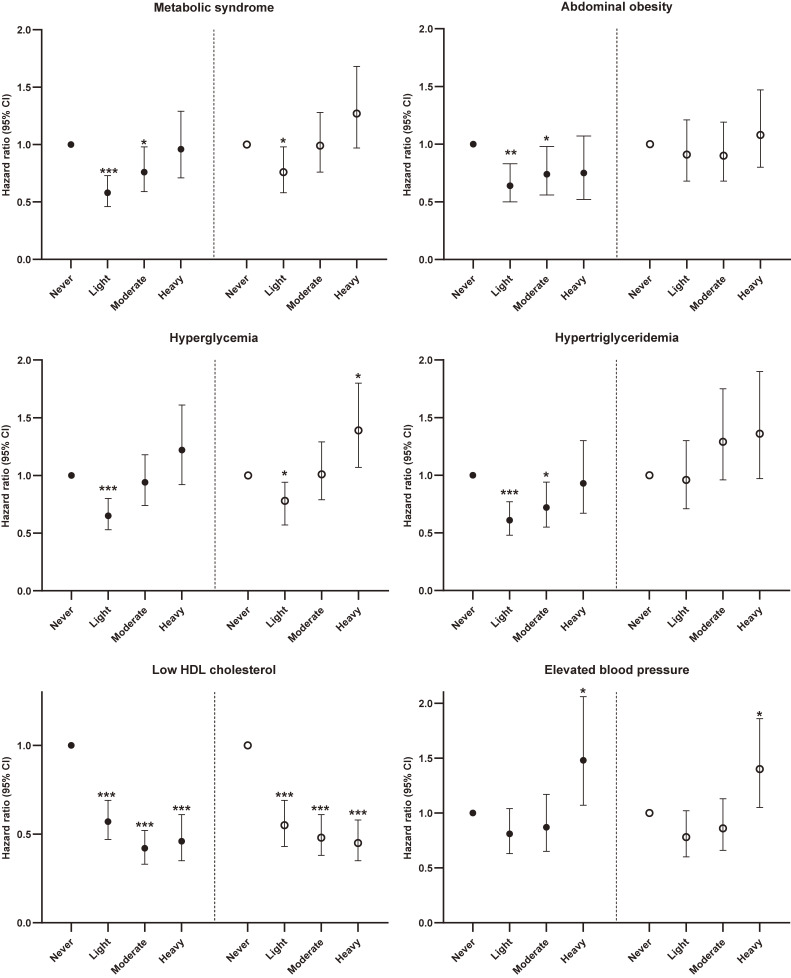
Hazard ratios (HRs) and 95% confidence intervals (CIs) for incident MetS and its components according to alcohol consumption by smoking status in men. Closed circles, non-smokers; open circles, current smokers. Multivariate Cox proportional hazards model was adjusted for age, residential location, household income, education level, physical activity, BMI, energy intake, fruit intake, meat intake, refined grain intake, whole grain intake, and dairy intake (^*^P < 0.05; ^**^P < 0.01; ^***^P < 0.0001 versus never drinkers). Alcohol consumption was never, light (0–15 g/day), moderate (15<–40 g/day), and heavy (>40 g/day). BMI, body mass index; MetS, metabolic syndrome.

Risk for MetS and its components according to alcohol consumption in men is presented by smoking status in Figure 1. In non-smokers, light or moderate alcohol consumption was inversely associated with MetS and its components including abdominal obesity, hyperglycemia, hypertriglyceridemia, and low HDL-C. Heavy alcohol consumption was associated with a higher risk of elevated blood pressure (HR 1.48; 95% CI, 1.07–2.06; *P* = 0.020) in men and abdominal obesity (HR 1.86; 95% CI, 1.06–3.27; *P* = 0.030) in women. Unlike non-smokers, no inverse association of light alcohol consumption with hypertriglyceridemia and abdominal obesity was seen in currently smoking men. Heavy alcohol consumption was associated with a higher risk of hyperglycemia in current smokers (HR 1.39; 95% CI, 1.07–1.80; *P* = 0.014). In particular, the strong interaction effect of alcohol consumption/smoking on hypertriglyceridemia was observed (Table 4).

**Table 4.** Hazard ratios and 95% confidence intervals for risk of incident hypertriglyceridemia according to alcohol consumption stratified by smoking status in Korean men

Alcohol consumption (g/day)					

	Never	Light (0–15)	Moderate (15<–40)	Heavy (>40)	*P* trend
**Non-smokers** (*n* = 1,120)					
*n* (*n* of cases)	283 (121)	490 (169)	238 (102)	109 (55)	
Model^a^	1	0.61 (0.48–0.77)	0.72 (0.55–0.94)	0.93 (0.67–1.30)	0.494
*P* value		<0.0001	0.018	0.673	
**Current smokers** (*n* = 1,010)					
*n* (*n* of cases)	156 (61)	383 (158)	299 (160)	172 (88)	
Model	1	0.96 (0.71–1.30)	1.29 (0.96–1.75)	1.36 (0.97–1.90)	0.005
*P* value		0.795	0.094	0.073	
*P* for interaction		0.0057	<0.0001	<0.0001	

^a^Model was adjusted for age, residential location, household income, education level, physical activity, BMI, energy intake, fruit intake, meat intake, refined grain intake, whole grain intake, and dairy intake.

Risk for MetS and its components according to alcohol consumption by smoking duration in men is in eTable 1. Light alcohol consumption was associated with lower risk of MetS and its components including abdominal obesity, hyperglycemia, hypertriglyceridemia, and low HDL-C regardless of smoking duration. Heavy alcohol consumption was associated with higher risk of hyperglycemia (HR 1.31; 95% CI, 1.04–1.65; *P* = 0.023) and elevated blood pressure (HR 1.53; 95% CI, 1.19–1.99; *P* = 0.001) among participants who had more than 20 years of smoking duration.

Additionally, risk for MetS and its components according to alcohol consumption was examined by residential location (data not shown) in men. Light alcohol consumption was inversely associated with MetS and all components in both rural and urban area. Heavy alcohol consumption was positively associated with elevated blood pressure in both areas, and with hyperglycemia in only urban area.

## DISCUSSION

This prospective study found that long-term light or moderate alcohol consumption was associated with lower risk of incident MetS, abdominal obesity, hyperglycemia, hypertriglyceridemia, and low HDL-C, and heavy alcohol consumption was associated with higher risk of incident elevated blood pressure for men, and abdominal obesity for women in non-smokers. Unlike non-smoking men, no inverse association of light or moderate alcohol consumption with hypertriglyceridemia and abdominal obesity was seen, whereas a positive association between heavy alcohol consumption and hyperglycemia was observed in smoking men. These results suggest that the association between long term alcohol consumption and risk of developing MetS and its components is substantially affected by smoking status.

Our findings on the association between alcohol intake and MetS or its components are in line with previous studies in white populations. The Quebec Cardiovascular study, during 13 years of follow-up, reported that one drink (<15.2 g/day) was associated with 43% lower risk of developing MetS.^27^ A longitudinal study among an Italian population aged ≥65 years showed that heavy alcohol consumption (>48 g/day for men, >24 g/day for women) was associated with an increased risk of abdominal obesity, hyperglycemia, and high blood pressure, but not with MetS in men.^28^

Light or moderate alcohol consumption was associated with lower risk of hypertriglyceridemia in non-smoking men. The beneficial association of alcohol may be explained by the effect of ethanol to decrease TG concentration through the increased activity of lipoprotein lipase.^29^ A study among an American older population showed significantly lower TG concentrations in individuals who consumed one or two drinks per day compared to non-drinkers.^30^ However, the inverse relationship between long term alcohol consumption and hypertriglyceridemia was not apparent in currently smoking men. This finding suggest that smoking strongly modifies the relationship between alcohol drinking and TG concentration. Similarly, a cross-sectional study reported that smoking accentuated the elevating effect of heavy alcohol drinking on TG in a male Chinese population suggests strong interactions between alcohol drinking and cigarette smoking on lipid profiles.^15^ Smoking also affects lipoprotein lipase activity, so it is involved in TG metabolism.^31^ Previous studies reported that smokers had significantly higher prevalence of hypertriglyceridemia than non-smokers.^32^^,^^33^

Smoking is known to be a risk factor for abdominal visceral fat accumulation.^34^ This effect could explain the lack of the protective association of light or moderate alcohol consumption on abdominal obesity in smoking men. Moreover, abdominal visceral fat is strongly associated with higher serum TG concentrations.^35^ Additionally, the effect of smoking on increasing abdominal obesity may be related to cortisol. Nicotine from cigarette smoking elevates cortisol level,^36^ which may increase fat storage in abdominal or visceral deposit.^37^ A cross sectional study of British population aged 45–79 years reported that current smokers had higher waist circumference than never smokers.^38^ Given these results, smoking status should be considered in the analysis on the association between alcohol consumption and lipid metabolism.

On the other hand, heavy alcohol consumption was associated with greater risk of hyperglycemia in current smokers. This phenomenon may be related to insulin resistance. Alcohol induces insulin resistance through abnormalities in signal transduction for glucose uptake.^39^ A recent study reported that alcohol abstinence significantly reduces fasting blood glucose levels and increases insulin sensitivity in Japanese men who routinely drank alcohol.^40^ Smoking may aggravate the harmful effects of alcohol intake on glucose level and insulin resistance because smoking itself is an independent risk factor for development of insulin resistance and type 2 diabetes.^41^ A prospective study with a 5 year follow-up among Japanese men showed an increase in fasting glucose level in current smokers that was significantly higher than never smokers.^42^ An intervention study found that smokers were less insulin sensitive compared with controls and insulin sensitivity increased after either 1 or 2 weeks of smoking cessation. This result indicates nicotine in tobacco smoke induces insulin resistance in human adults.^43^ Kim et al reported that length of smoking cessation period independently predicts insulin resistance in Korean male ex-smokers.^44^ In our study, smoking diminished the beneficial effects of light alcohol consumption on MetS and its components, such as hypertriglyceridemia and abdominal obesity, and deteriorated the unfavorable effect of heavy alcohol consumption on MetS components, such as hyperglycemia.

This study has several strengths. To the best of our knowledge, this is the first study to examine how smoking influences the relation of long-term alcohol consumption on risk of MetS and its components using data from a large population. Also, chronic alcohol consumption was accurately estimated from habitual intake using the average value from repeated measures during a long follow-up (about 10 years). The study adjusted for most major confounders for MetS and its components, including lifestyle and dietary factors. In addition, standardized protocols were used to obtain data on exposure and outcome.

However, this study had some limitations. The association between chronic alcohol consumption and MetS and its components in women might be diminished due to the small number of female heavy alcohol drinkers. There is the possibility that the results could be biased because of loss to follow-up. Although the study adjusted for major confounding factors, residual or unmeasured confounding factors on the association between alcohol consumption and MetS are possible.

In conclusion, long-term alcohol consumption has both beneficial and detrimental effects on MetS and it components depending on the amount of alcohol consumed, and the association is importantly affected by smoking status. Therefore, smoking status should be taken into account in the guideline for alcohol consumption for prevention and management of chronic diseases, including MetS.

## APPENDIX A. SUPPLEMENTARY DATA

eTable 1. The Hazard ratios (HRs) and 95% confidence intervals (CIs) for risk of incident metabolic syndrome components according to alcohol consumption stratified by smoking duration in men^a^

eFigure 1. Flowchart of study population. HDL-C, high density lipoprotein cholesterol.
