# Supplementary material for: The Effect of Smoking and Sex on the Association Between Long-term Alcohol Consumption and Metabolic Syndrome in a Middle-aged and Older Population
Source: J Epidemiol. 2021 Apr 5;31(4):249–58. doi: 10.2188/jea.JE20190328 (PMC7940979; doi:10.2188/jea.JE20190328)
Supplement: Supplementary file 1 [file je-31-249-s001.pdf]

eTable 1. The Hazard ratios (HRs) and 95% confidence intervals (CIs) for risk of incident metabolic syndrome components according to alcohol consumption stratified by smoking duration in men[a]

	Alcohol consumption (g/day)				
	Never	Light (0–15)	Moderate (15<–40)	Heavy (>40)	*P*–trend
Abdominal obesity (*n*=3,026)					
Never (*n*=593)	1.00	0.65 (0.45–0.94)	0.79 (0.49–1.26)	1.40 (0.73–2.67)	0.969
<20 y (*n*=467)	1.00	0.86 (0.44–1.65)	1.01 (0.51–2.01)	0.80 (0.37–1.75)	0.914
≥20 y (*n*=1,966)	1.00	0.71 (0.56–0.91)	0.75 (0.59–0.96)	0.87 (0.66–1.14)	0.565
Hyperglycemia (*n*=2,918)					
Never (*n*=601)	1.00	0.64 (0.46–0.87)	1.02 (0.70–1.50)	1.16 (0.70–1.92)	0.376
<20 y (*n*=450)	1.00	0.54 (0.35–0.82)	0.62 (0.40–0.98)	1.16 (0.70–1.92)	0.092
≥20 y (*n*=1,867)	1.00	0.71 (0.58–0.88)	0.99 (0.80–1.23)	1.31 (1.04–1.65)	<0.0001
Hypertriglyceridemia (*n*=2,204)					
Never (*n*=463)	1.00	0.66 (0.45–0.95)	0.84 (0.53–1.33)	1.45 (0.81–2.61)	0.493
<20 y (*n*=314)	1.00	0.91 (0.50–1.65)	0.75 (0.40–1.43)	1.03 (0.51–2.07)	0.551
≥20 y (*n*=1,336)	1.00	0.69 (0.54–0.87)	0.98 (0.77–1.26)	0.96 (0.73–1.28)	0.166

Low HDL-C (*n*=2,864)					
Never (*n*=580)	1.00	0.71 (0.54–0.93)	0.44 (0.30–0.63)	0.56 (0.34–0.91)	<0.0001
<20 y (*n*=442)	1.00	0.52 (0.34–0.82)	0.38 (0.23–0.62)	0.33 (0.19–0.59)	0.001
≥20 y (*n*=1,842)	1.00	0.50 (0.41–0.60)	0.44 (0.36–0.54)	0.44 (0.35–0.55)	<0.0001
Elevated blood pressure (*n*=2,017)					
Never (*n*=375)	1.00	0.96 (0.67–1.37)	1.27 (0.80–2.02)	1.56 (0.79–3.10)	0.155
<20 y (*n*=317)	1.00	0.73 (0.44–1.22)	0.63 (0.36–1.10)	1.20 (0.65–2.21)	0.495
≥20 y (*n*=1,325)	1.00	0.78 (0.62–0.98)	0.88 (0.69–1.13)	1.53 (1.19–1.99)	0.0001
MetS (*n*=2,866)					
Never (*n*=583)	1.00	0.65 (0.46–0.90)	0.77 (0.50–1.19)	0.95 (0.54–1.67)	0.486
<20 y (*n*=442)	1.00	0.64 (0.37–1.11)	0.71 (0.40–1.28)	0.71 (0.37–1.37)	0.682
≥20 y (*n*=1,841)	1.00	0.65 (0.52–0.81)	0.90 (0.72–1.12)	1.18 (0.93–1.50)	0.002

HDL-C, high density lipoprotein cholesterol; MetS, metabolic syndrome.

[a]HRs and 95% CIs were adjusted for age, residential location, household income, education level, physical activity, body mass index, energy intake, fruit intake, meat intake, refined grain intake, whole grain intake, and dairy intake. Subjects whose drinking period was missing were excluded from the analysis; Abdominal obesity (*n*=14), hyperglycemia (*n*=23), hypertriglyceridemia (*n*=17), low HDL-C (*n*=23), elevated blood pressure (*n*=14), metabolic syndrome (*n*=18).

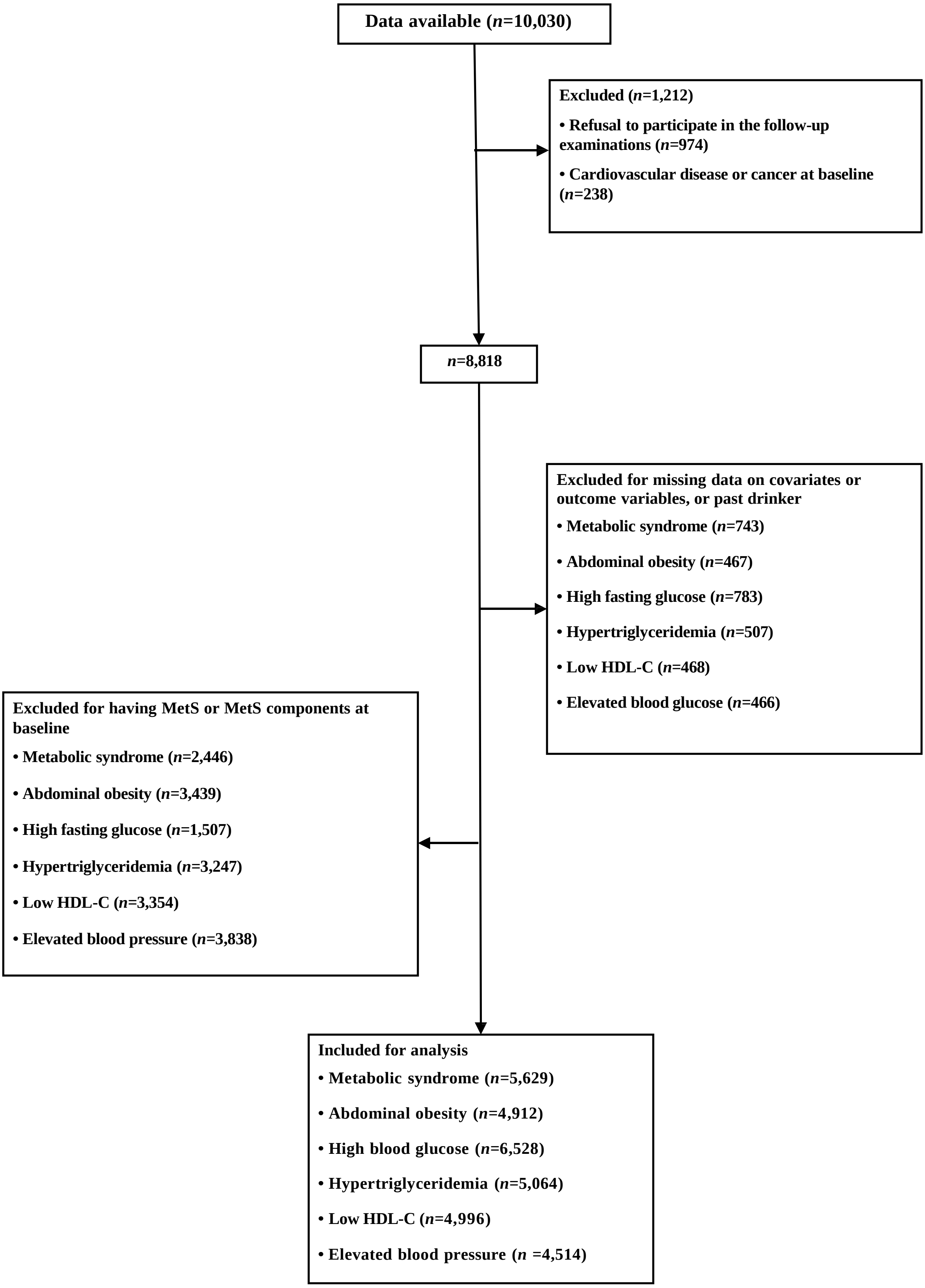

eFigure 1. Flowchart of study population. HDL-C, high density lipoprotein cholesterol.
